# Supplementary material for: Stereocomplexation of Poly(Lactic Acid)s on Graphite Nanoplatelets: From Functionalized Nanoparticles to Self-assembled Nanostructures
Source: Front Chem. 2019 Mar 29;7:176. doi: 10.3389/fchem.2019.00176 (PMC6450084; doi:10.3389/fchem.2019.00176)
Supplement: Supplementary file 1 [file Data_Sheet_1.pdf]

SUPPORTING INFORMATION

Stereocomplexation of Poly(Lactic Acid)s on Graphite Nanoplatelets: from Functionalized Nanoparticles to Self-Assembled Nanostructures.

Matteo Eleuteri, Mar Bernal, Marco Milanesio, Orietta Monticelli, Alberto Fina

1.1 Characterization of Pyr-L

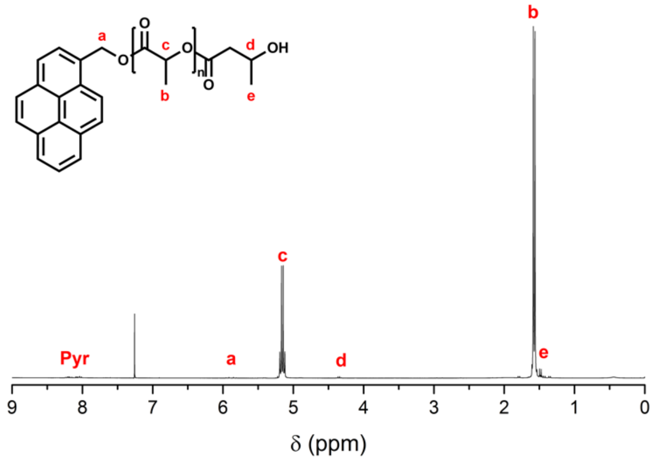

Figure S1. ^{1}H NMR spectrum in $CDCl_3$ of Pyr-L.

The UV-Vis spectra of Pyr-L in DMF at a concentration of 0.5 mg mL^{-1} is shown in Figure S2. The typical absorbance bands of pyrene in the region of 270 – 350 nm can be clearly observed.

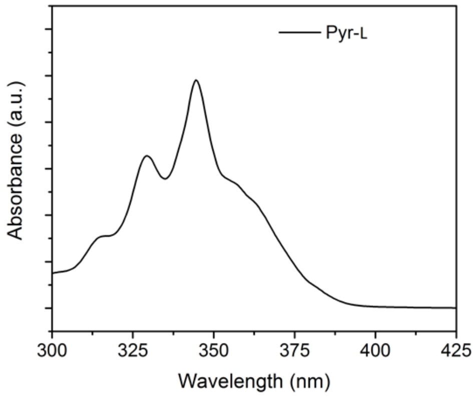

Figure S2. UV-Vis absorption spectra of Pyr-L in DMF at a concentration of 0.5 mg mL^{-1}.

1.2 Effect of the Solvent on the Pyr-L/Pyr-D Stereocomplexation

Pyr-L/Pyr-D stereocomplexes were prepared from solutions in different solvents, namely chloroform and N,N-dimethylformamide. The first is a conventional solvent for polylactic acid, whereas the second was selected for its well-known affinity to GnP. While sterecomplexation is observed to occur in both solvents, results from thermal analysed as well as spectroscopy evidence for dramatic differences in organization of SC crystallites, as a function of the PLA oligomers with the solvent.

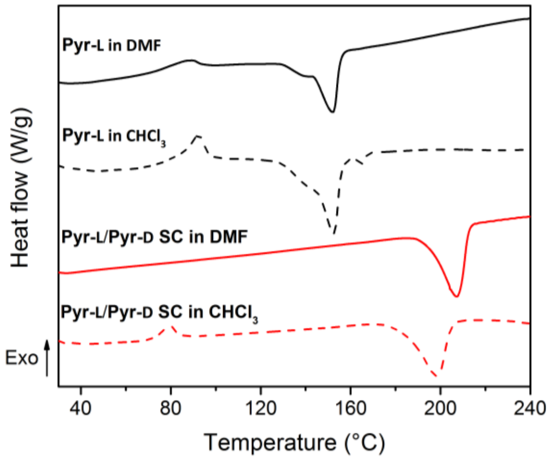

Figure S3. DSC thermograms of Pyr-L and Pyr-L/Pyr-D SC, recrystallized from DMF and $CHCl_3$, recorded during the second heating (10 °C min^{-1}).

Table S1. Thermal parameters of Pyr-L and Pyr-L/Pyr-D SC obtained after crystallization in DMF and $CHCl_3$: cold crystallization temperature (T_{cc}) and enthalpy (ΔH_{cc}), melting temperature (T_m) and enthalpy (ΔH_m) and temperature corresponding to the maximum DTG peaks (T_{max}) obtained from the first derivative curve of the TGA thermograph of Pyr-L and Pyr-L/Pyr-D SC.

Sample	T_{cc} [a] (°C)	ΔH_{cc} [a] (J g^{-1})	T_m [a] (°C)	ΔH_m [a] (J g^{-1})	T_{max} [b] (°C)
**Pyr-L (DMF)**	87.1	7.3	138.5, 152.2	39.3	264.2
**Pyr-L ($CHCl_3$)**	92.2	11.0	140.6, 152.5, 165.1	38.8	274.0
**Pyr-L/Pyr-D SC (DMF)**	-	-	207.5	45.1	296.6
**Pyr-L/Pyr-D SC ($CHCl_3$)**	79.3	5.6	198.7	43.5	293.4

[a] Determined by DSC analysis, second heating (10 °C min^{-1}).
[b] Determined by TGA analysis.

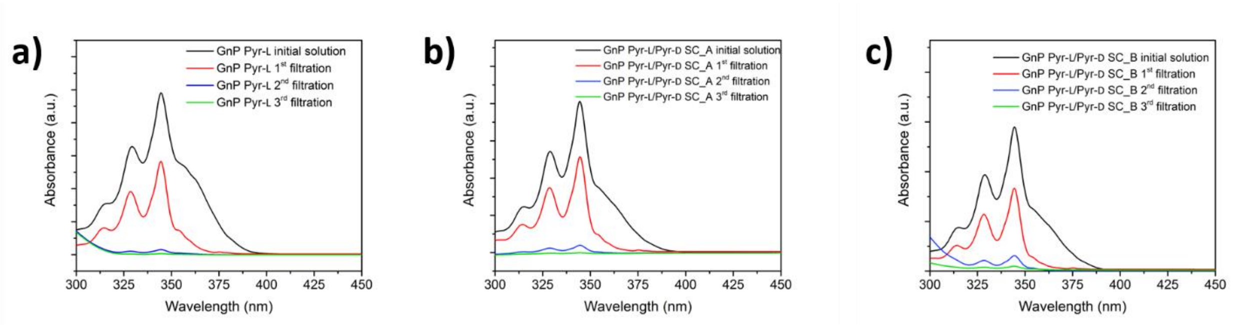

Figure S4. UV-Vis spectra obtained after dispersion-filtration cycles during the preparation of a) GnP Pyr-L, b) GnP Pyr-L/Pyr-D SC_A and c) GnP Pyr-L/Pyr-D SC_B.

1.3 XPS analysis

Pyr-L and Pyr-L/Pyr-D SC were first measured as reference samples in order to facilitate the interpretation of the XPS spectra of supramolecularly functionalized GnP (Figure S5). In the case of Pyr-L and Pyr-L/Pyr-D SC, the C_{1s} spectra show the components related to the polymers structure:(Shakesheff et al., 1997; Jordá-Vilaplana et al., 2014) the peak at ~ 284.9 eV attributed to aliphatic carbons (-CH_2, -CH_3), the band at 285.5 eV (C-OH), the C-O-C peak at 286.8 eV and the 288.9 eV binding energy of the -C=O. The deconvolution of the C_{1s} spectra of the functionalized GnP are dominated by the peak at 284.4 eV, related to the sp^2 C-C bonds and thus the differentiation of the aliphatic carbons of the polymer backbone remains difficult, suggesting for a limited fraction of PyrL or PyrD onto GnP. The O_{1s} spectra (Figure 5b) of Pyr-L and Pyr-L/Pyr-D SC were deconvoluted into the three bands typical of the composition of PLA: the O-C=O at 531.5 eV, the C-O-C at 533 eV and the C-OH at 534.2 eV. On the other hand, the O_{1s} spectrum of GnP showed only two components due to the C=O at 530.5 eV and a broad band related to the C-O-C group. The successful functionalization of the GnP with the polymer chains is confirmed by the presence on the O_{1s} spectra of the corresponding oxygen bands observed in the O_{1s} spectra of Pyr-L and Pyr-L/Pyr-D SC. The values of the at.% of each oxygen functional group are summarized in Table S2. Atomic ratios (at.%) were calculated from experimental intensity ratios and normalized by atomic sensitivity factors. The C_{1s} and O_{1s} peaks were fitted as following the previously described procedure.(Bernal et al.)

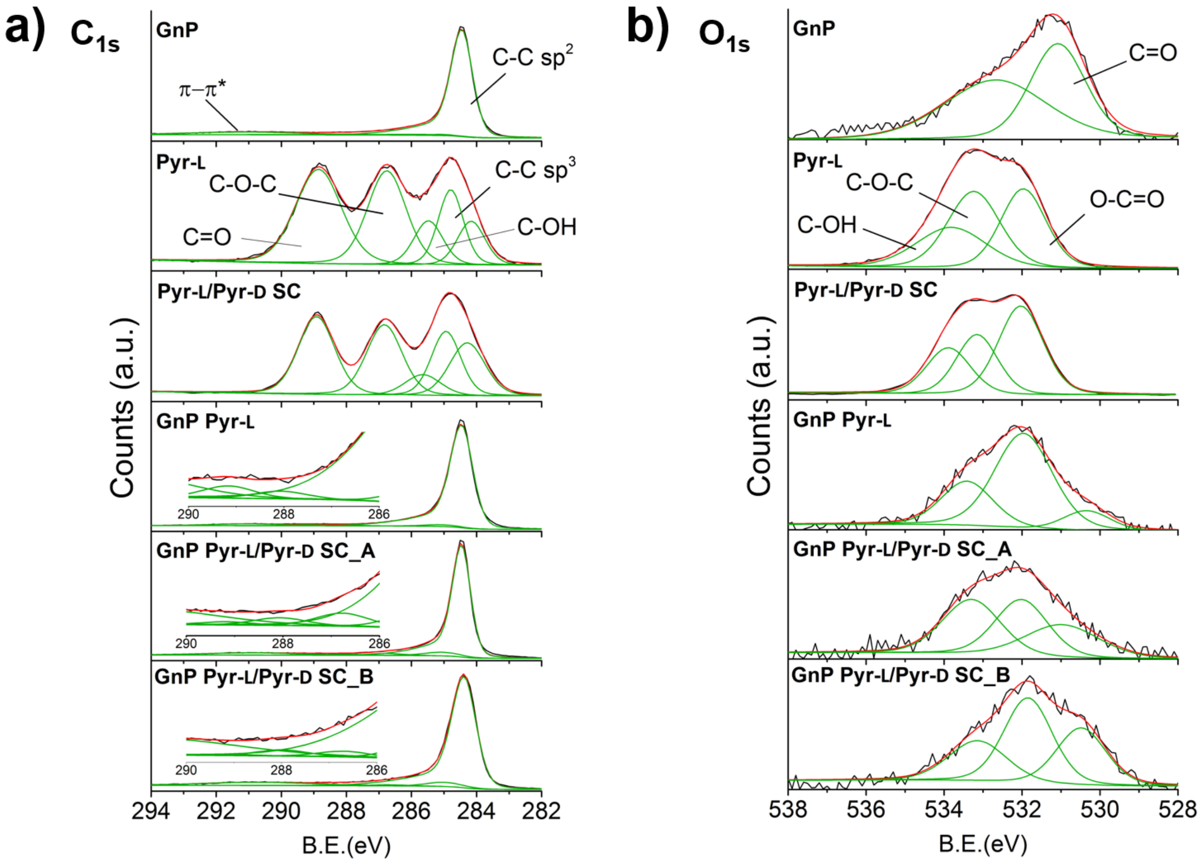

Figure S5. C_{1s} and O_{1s} XPS spectra of GnP, Pyr-L, Pyr-L/Pyr-D SC_A, Pyr-L/Pyr-D SC_B, GnP Pyr-L, GnP Pyr-L/Pyr-D SC_A and GnP Pyr-L/Pyr-D SC_B.

Table S2. XPS peak assignment and atomic percentage (at.%) for Pyr-L, Pyr-L/Pyr-D SC, GnP, GnP Pyr-L, GnP Pyr-L/Pyr-D SC _A and GnP Pyr-L/Pyr-D SC _B.

**Sample**	**Total C_{1s} at.%**	**Total O_{1s} at.%**	**Contributions from O to C_{1s} spectra at.%**		
			**C-OH**	**C-O-C**	**C=O**
**Pyr-L**	59.2	40.8	6.1	26.1	20.7
**Pyr-L/Pyr-D SC**	59.2	40.8	4.5	22.7	17.0
**GnP**	96.5	3.5	0.9	0.8	1.8
**GnP Pyr-L**	96.4	3.6	0.9	1.1	1.5
**GnP Pyr-L/Pyr-D SC _A**	95.5	4.5	0.6	3.3	1.2

**GnP Pyr-L/Pyr-D SC _B**	96.0	4.0	0.8	1.5	1.8

1.4 Adsorption concentration and adsorption density

The adsorption concentration and density of the stereoisomers and the SCs were determined from the TGA data of the fuctionalized GnP.

The adsorption concentration (mmol g^{-1}) was calculated from the mmol of polymer chains adsorbed per gram of framework graphene carbon according to the literature:(Hu et al., 2017)

$$GC\ (\frac{mmol}{g}) = \frac{\left({}^{M_p}\!/_{(M_p + M_G)} \Big/ M_{wp} \right)}{\left(1 - {}^{M_p}\!/_{(M_p + M_G)} \right)} \times 1000$$

where M_p is the total mass of adsorbed polymer, M_G is the total mass of GnP and M_{wp} is the molecular weight of the polymer in g mol^{-1}.

The adsorption density, defined by the number of polymers per area of GnP surface, *N/A*, was calculated as:(Chadwick et al., 2013)

$$\frac{N}{A} = \frac{M}{A}\frac{M_p}{M_G}\frac{1}{M_{wp}}$$

where *M/A* is the mass per unit area of graphene estimated as 7.48 x 10^{-7} kg m^{-2} (assuming an average area of GnP of 100 μm^2 and a density of 2200 kg m^{-3}) and M_{wp} is converted to kg $molecule^{-1}$.

1.5 Additional characterization of GNP Pyr-L/Pyr-D SC nanopapers

Thermogravimetry was used to assess the content of organic fraction, namely the Pyr-l/Pyr-D SC in the nanopapers prepared form different enantiomers concentrations. Results are reported in Figure S6.

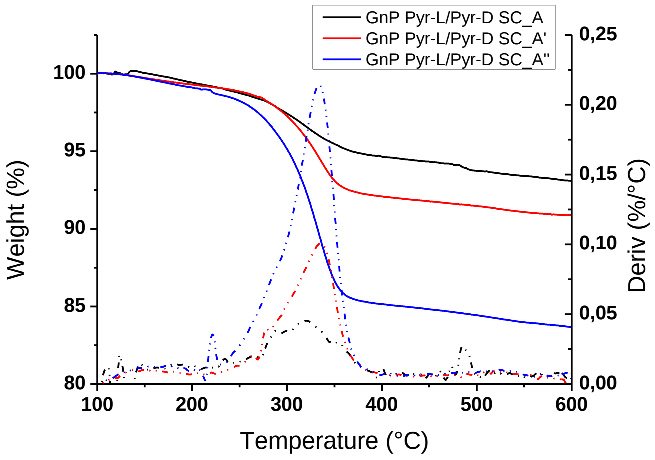

Figure S6. Thermogravimetric plots for nanopapers of GnP Pyr-L/Pyr-D SC prepared with different enantiomers concentrations

2D XRD were reduced to intensity vs. 2θ profiles, to investigate preferential orientations in the nanopapers and well as to obtain averaged XRD profiles over the whole possible orientations. Results are reported in Figure S7.

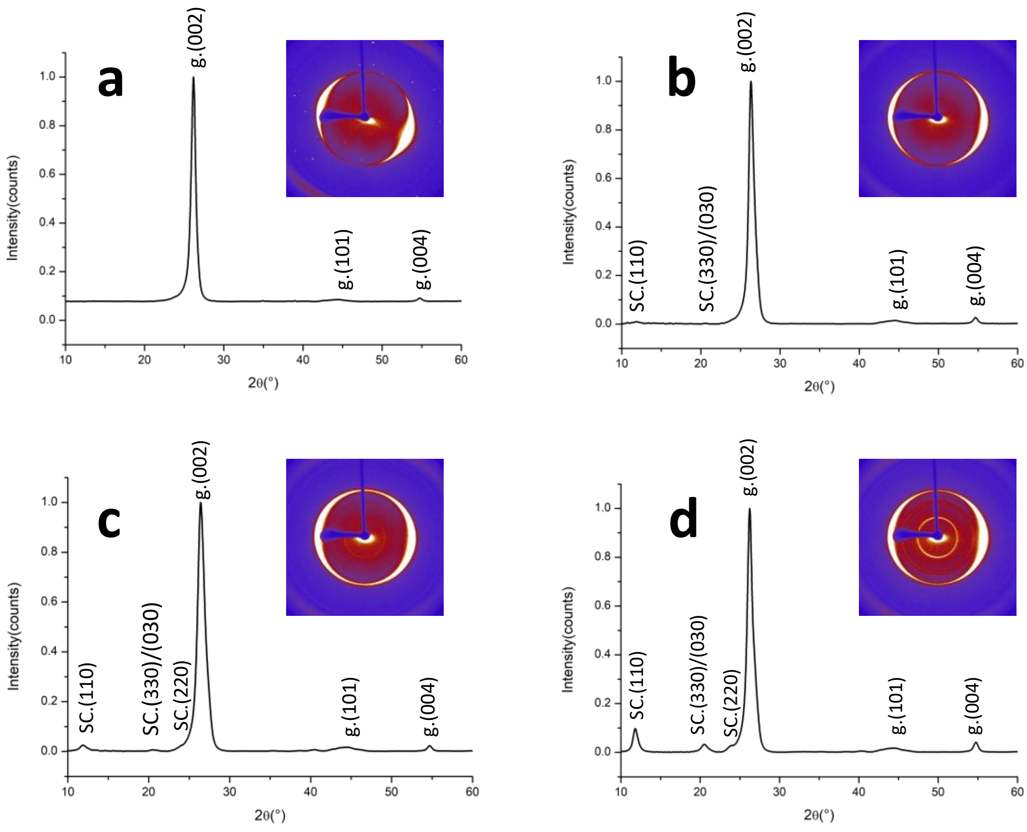

Figure S7. XRD spectra from 0-360° intergration of 2D Patterns obtained on nanopapers of pristine GnP (a) GnP Pyr-L/Pyr-D SC_A (b), GnP Pyr-L/Pyr-D SC_A′ (c) and GnP Pyr-L/Pyr-D SC_A″ (d). Peaks are identified with their crystalline plane s assignment graphene (g) or stereocomplex (SC)

References

Bernal, M.M., Di Pierro, A., Novara, C., Giorgis, F., Mortazavi, B., Saracco, G., et al. Edge-Grafted Molecular Junctions between Graphene Nanoplatelets: Applied Chemistry to Enhance Heat Transfer in Nanomaterials. *Advanced Functional Materials*, 1706954-n/a. doi: 10.1002/adfm.201706954.

Chadwick, R.C., Khan, U., Coleman, J.N., and Adronov, A. (2013). Polymer Grafting to Single-Walled Carbon Nanotubes: Effect of Chain Length on Solubility, Graft Density and Mechanical Properties of Macroscopic Structures. *Small* 9(4), 552-560. doi: doi:10.1002/smll.201201683.

Hu, S., Laker, Z.P.L., Leese, H.S., Rubio, N., De Marco, M., Au, H., et al. (2017). Thermochemical functionalisation of graphenes with minimal framework damage. *Chemical Science* 8(9), 6149-6154. doi: 10.1039/C6SC05603B.

Jordá-Vilaplana, A., Fombuena, V., García-García, D., Samper, M.D., and Sánchez-Nácher, L. (2014). Surface modification of polylactic acid (PLA) by air atmospheric plasma treatment. *European Polymer Journal* 58, 23-33. doi: https://doi.org/10.1016/j.eurpolymj.2014.06.002.

Shakesheff, K.M., Evora, C., Soriano, I., and Langer, R. (1997). The Adsorption of Poly(vinyl alcohol) to Biodegradable Microparticles Studied by X-Ray Photoelectron Spectroscopy (XPS). *Journal of Colloid and Interface Science* 185(2), 538-547. doi: https://doi.org/10.1006/jcis.1996.4637.
